# Detachment from Work: A Diary Study on Telepressure, Smartphone Use and Empathy

**DOI:** 10.5334/pb.477

**Published:** 2019-06-27

**Authors:** Ruben Cambier, Daantje Derks, Peter Vlerick

**Affiliations:** 1 Department of Personnel Management, Work and Organizational Psychology, Ghent University, Ghent, BE; 2 Department of Work and Organizational Psychology, Erasmus University Rotterdam, Rotterdam, NL

**Keywords:** diary study, empathy, psychological detachment, smartphone use, telepressure

## Abstract

Technology has drastically reshaped the workplace over the past decades. While it provides organizations and their employees a variety of benefits, there is also a growing perception that technological advancements (e.g., the evolution from telephone to smartphone) in the workplace may have a negative impact on employees’ mental health. Using a diary approach, we examined the direct effect of workplace telepressure during off-job hours on psychological detachment from work and the potential mediating role of work-related smartphone use during off-job hours in this relation. In addition, employees’ individual differences in empathy was proposed to act as a cross-level moderator of the relation between workplace telepressure and work-related smartphone use. A sample of 80 employees, representing a wide range of occupations and organizations, completed a daily survey on five successive workdays (*N* = 337–400 day-level observations). Results of multilevel analyses yielded no direct effect of workplace telepressure on psychological detachment on a day-to-day basis. Yet, the results supported a negative indirect effect of daily workplace telepressure during off-job hours on daily psychological detachment, mediated via daily work-related smartphone use during off-job hours. Additionally, the relation between workplace telepressure and work-related smartphone use was not strengthened by the affective component nor the cognitive component of other-oriented empathy. Our study highlights the importance of a clear organizational policy regarding work-related smartphone use during off-job hours and provides valuable input for strategies aiming to ameliorate employees’ psychological detachment and proper smartphone use.

New information communication technology (ICT) devices such as smartphones have enabled employees to stay connected to their work twenty-four hours a day (Härmä, 2006; Towers, Duxbury, Higgings, & Thomas, 2006). Accordingly, expectations concerning employees’ responsiveness towards work-related ICT messages have amplified remarkably (Mazmanian, Yates, & Orlikowski, 2006). Therefore, employees might feel the need to respond to incoming work-related ICT messages in a timely manner, regardless of their regular work schedule (Barber & Santuzzi, 2015; Davis, 2002). However, work-related ICT use during off-job hours might impede employees’ recovery from work (e.g., Derks, Van Mierlo, & Schmitz, 2014; Schlachter, McDowall, Cropley, & Inceoglu, 2018; Van Laethem, van Vianen, & Derks, 2018).

Moreover, not only ICT use for work purposes during off-job hours such as replying to work-related messages, but also the preoccupation with and urge for sending those replies quickly (i.e., workplace telepressure) have been negatively associated with a highly powerful recovery experience: psychological detachment (Barber & Santuzzi, 2015; Grawitch, Werth, Palmer, Erb, & Lavigne, 2018; Santuzzi & Barber, 2018). Employees who reported higher levels of workplace telepressure seemed to have more trouble to mentally disconnect from work during off-job time. However, it is important to note that the strength of bivariate findings was meager (Barber & Santuzzi, 2015; Grawitch et al., 2018; Santuzzi & Barber, 2018) and workplace telepressure did not explain incremental variance in predicting psychological detachment when taking individual differences and work factors into account (Grawitch et al., 2018).

A possible explanation for the magnitude of these results might be the too generic assessment of workplace telepressure in previous research. As research on the stability of telepressure is limited and results are indefinite (Barber & Santuzzi, 2017; Santuzzi & Barber, 2018), it remains unclear whether the experience of workplace telepressure fluctuates from day to day or even throughout a single workday. Yet intuitively plausible, employees’ levels of telepressure concerning work-related ICT messages might differ between work and nonwork hours. Since, by definition, psychological detachment occurs during off-job hours (Sonnentag & Bayer, 2005), it is crucial and more ecologically valid to assess workplace telepressure during off-job hours when investigating the association between workplace telepressure and a recovery experience such as psychological detachment from work (Judge & Kammeyer-Mueller, 2012).

The present study seeks to contribute to existing ICT and work recovery literature by providing further insight into the relation between workplace telepressure and psychological detachment. Firstly, given that previous research did not differentiate between workplace telepressure during work and nonwork hours (Barber & Santuzzi, 2015; Grawitch et al., 2018; Santuzzi & Barber, 2018), we examined whether workplace telepressure during off-job hours obstructed psychological detachment from work.

Secondly, considering smartphones being the most ubiquitous ICT device nowadays (Steemers et al., 2017), we specifically investigated work-related smartphone use during off-job hours as a potential explanatory mechanism or mediator in this relation, shedding light on how workplace telepressure might influence psychological detachment.

Lastly, our study examined if individual differences in empathy played an enhancing role in the relation between workplace telepressure during off-job hours and work-related smartphone use during off-job hours. Feelings of empathy are not only restricted to face-to-face communication, it is also possible to have empathy through computer-mediated communication (Carrier, Spradline, Bunce, & Rosen, 2015). Empathic recipients might understand the sender’s point of view that having to linger on a reply can be bothersome for the sender whom awaits a response. Consistent with research on empathy-based helping (Eisenberg, Fabes, & Spinrad, 2006), this concern might reinforce the urge to respond to the message in a timely manner. The conceptual model of our study is outlined in Figure 1.

**Figure 1:**
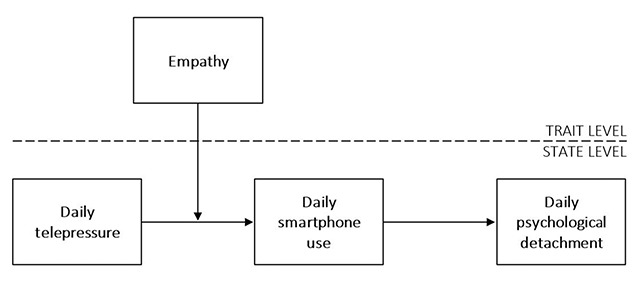
Conceptual model.

Because work stressors and recovery levels have shown to be dynamic and to fluctuate from one day to another (Ohly, Sonnentag, Niessen, & Zapf, 2010), we tested our propositions on a daily basis by conducting a five-day quantitative diary study using a mixed-subject research design. Hence, by capturing within-person fluctuations of workplace telepressure during off-job hours at day-level, we expand the existing base of knowledge regarding the within-person fluctuations within telepressure over time that has been heretofore exclusively limited to month-level time lags (Barber & Santuzzi, 2017; Santuzzi & Barber, 2018).

## Workplace telepressure and psychological detachment

Prior research in occupational health psychology described the importance and necessity of employee well-being in today’s workplace. Maintenance of good employee mental health contributes to enhanced business productivity along with a reduction in organizational costs due to decreases in absenteeism and staff turnover (see Harter, Schmidt, & Keyes, 2003, for a review). However, employee well-being tends to become impaired when employees are unable to properly detach from work and job-related issues outside of their regular work hours (Golden & Wiens-Tuers, 2008). Indeed, psychological detachment is considered to play a crucial role in the necessary processes to appropriately recover from work-induced tiredness after a working day or after demanding workdays (Etzion, Eden, & Lapidot, 1998; Sonnentag & Bayer, 2005). According to and consistent with the effort-recovery theory (Meijman & Mulder, 1998), the introduction of ICT devices into the workplace has made psychological detachment and, therefore, work recovery more difficult as employees can remain connected to their work twenty-four hours a day and job stressors may prolong or reoccur during off-job hours (e.g., Derks et al., 2014; Schlachter et al., 2018; Van Laethem et al., 2018).

Focusing on workplace settings, Barber and Santuzzi (2015) coined the term telepressure and defined its work-related experience as a psychological state consisting of the preoccupation and urge to quickly respond to work-related messages transmitted via ICT devices. Previous studies found that ICT-related cognition such as workplace telepressure has the potential to obstruct detachment from work (Barber & Santuzzi, 2015; Grawitch et al., 2018; Santuzzi & Barber, 2018). Although researchers have expended considerable effort to identify the impact of workplace telepressure on psychological detachment, they all focussed on workplace telepressure in general and measured it regardless of time of day. As work recovery processes occur mainly during off-job hours and workplace telepressure might substantially differ between work and nonwork hours, the present study specifically investigates workplace telepressure during off-job hours to adequately examine its impact on detachment from work. Based on past findings and the effort-recovery theory, we assume that on days when employees experience higher levels of workplace telepressure during off-job hours, they are less able to detach from work and job-related issues within the same day. This leads to our first hypothesis:

*Hypothesis 1: Daily workplace telepressure during off-job hours is negatively related to daily psychological detachment*.

## Workplace telepressure and work-related smartphone use

Over the past few years, the smartphone has gently been evolving into the most preferred and used ICT device (Steemers et al., 2017). Beyond mobile phones’ traditional voice call and SMS functionalities, smartphones can connect to the Internet and have an operating system capable of running downloaded applications. This opened doors to features that until then were exclusively for computers such as having access to a wide variety of communication channels. Today, voice call and SMS have lost popularity, the newly preferred communication channels on the smartphone are instant messaging and email (Steemers et al., 2017).

Previous research has shown that employees who experienced high levels of workplace telepressure also reported increased email responding (Barber & Santuzzi, 2015; Grawitch et al., 2018) and decreased response times on those emails (Barber & Santuzzi, 2015). Given the fact that a vast majority of individuals check incoming (work) emails via their smartphone on a daily basis (Steemers et al., 2017), it is plausible to expect a relation between workplace telepressure and work-related smartphone use during off-job hours. ICT messages received during off-job hours might be first noticed on the smartphone by popup notifications, as this ICT device is used most intensively and usually within arm’s reach. Due to convenience and improper time management, recipients might respond to these messages via the same ICT device. Indeed, prior research found that employees who experienced high workplace telepressure also reported increased work-related smartphone use during off-job hours (Van Laethem et al., 2018). In line with previous findings, we expect that on days when employees experience higher levels of workplace telepressure during off-job hours, they will more easily give in to their desire and urge to respond to work-related ICT messages. We formulate the following hypothesis:

*Hypothesis 2: Daily workplace telepressure during off-job hours is positively related to daily work-related smartphone use during off-job hours*.

## Work-related smartphone use and psychological detachment

According to the effort-recovery theory (Meijman & Mulder 1998), specific types of off-job activities may be harmful to powerful recovery experiences such as psychological detachment from work. Specifically, by performing work-related activities after work by means of ICT devices in general (Park, Fritz, & Jex, 2011; Schlachter et al., 2018) and by the smartphone in particular (Derks et al., 2014; Van Laethem et al., 2018), the employee stays psychologically occupied with work and corresponding job stressors remain present. To that end, we assume that on days when employees constantly stay online and answer work-related messages with their smartphone after work hours comes at cost of employees’ psychological detachment within the same day, because the time spent on work takes time away to recover from job stressors. As such, we formulate the following hypothesis:

*Hypothesis 3: Daily work-related smartphone use during off-job hours is negatively related to daily psychological detachment*.

As previously mentioned, employees’ experience of workplace telepressure might interfere with their psychological detachment from work (Barber & Santuzzi, 2015; Grawitch et al., 2018; Santuzzi & Barber, 2018). However, so far, research into explanatory mechanisms or mediators in this relationship is non-existing. As high levels of workplace telepressure during off-job hours is expected to trigger more usage of smartphone during off-job time for work purposes (Hypothesis 2), and as this usage is expected to relate to a lack of psychological detachment (Hypothesis 3), we propose that daily workplace telepressure during off-job hours is negatively and indirectly related to daily psychological detachment, through the mediation of work-related smartphone use during off-job hours. In other words, the conversion of one’s intention to respond to a work-related ICT message into real action (e.g., checking, reading and/or replying on a message), is assumed to undermine psychological detachment from work. Therefore we hypothesize:

*Hypothesis 4: The relationship between daily workplace telepressure during off-job hours and daily psychological detachment is mediated by daily work-related smartphone use during off-job hours*.

## The moderating role of empathy

The experience of workplace telepressure was originally argued as being primarily driven by external pressures such as social norms around responsiveness towards computer-mediated communication (Barber & Santuzzi, 2015). However, Grawitch and colleagues (2018) found that a sizeable amount of explained variance in workplace telepressure was attributable to internally driven pressures such as neuroticism, self-control, and workaholism. To the best of our knowledge, prior studies exclusively examined individual differences as direct predictors of workplace telepressure and paid no attention to their potential moderating role on workplace telepressure and its outcomes.

Unless employees are required to be on-call by their organization, responding to work-related ICT messages during off-job hours can be considered as a prosocial action, as it may help others (i.e., the sender) and is carried out entirely voluntary (Eisenberg et al., 2006). An extensive body of research indicates that other-oriented empathy is an essential individual difference factor in predicting prosocial behaviour and effectiveness in the workplace and beyond (For a review, see Clark, Robertson, & Young, 2019; Eisenberg et al., 2006). Empathy broadly refers to the tendency to be psychologically aware of others’ perspectives and feelings (Decety & Lamm, 2006). Following the multidimensional approach (Konrath, O’Brien, & Hsing, 2011), other-oriented empathy consists of a cognitive (i.e., perspective taking) and an affective (i.e., empathic concern) component.

Although research has shown that employees tend to use their smartphone more for work purposes during off-job hours when they experience workplace telepressure (Van Laethem et al., 2018), it is possible that especially empathic employees use their smartphone more intensively for work purposes during off-job hours when experiencing high levels of workplace telepressure at that moment. Given that feelings of empathy are not restricted to face-to-face communication (Carrier et al., 2015), employees who receive a work-related message via their smartphone can also empathize with the sender of this message. Nowadays, expectations on responsiveness towards business communication are high and expectancy violations are often evaluated as unpleasant by the sender whom awaits a response (Kalman & Rafaeli, 2011). As such, an empathic recipient may adopt the sender’s perspective and realize that postponing replies can be bothersome and unpleasant for the sender, which may evoke a desire to help (Eisenberg et al., 2006). Hence, reinforcing the urge to timely respond to the message. We formulate the following hypothesis:

*Hypothesis 5: Empathy* (*empathic concern and perspective taking*) *moderates the positive relation between workplace telepressure during off-job hours and work-related smartphone use during off-job hours, such that the positive relation between workplace telepressure and work-related smartphone use during off-job hours will be stronger for employees high on empathy compared to employees low on empathy*.

## Method

### Sample and procedure

We tested our hypotheses with data from a sample recruited via two strategies: announcements posted on social network sites and invitation emails to personal contacts of the authors. Both actions had the request to participate in a five-day diary study on ‘work-related smartphone use during off-job hours’. Potential participants were asked to send an email to one of the researchers. Participants were required to be (a) over 18 years old, (b) in the possession of a smartphone, which they use for work-related purposes and (c) to work five successive days during the workweek the data was acquired. At any time, respondents were free to decide whether to withdraw from the study. Participation did not yield any reward.

The data were collected via online surveys using a diary-study approach in which respondents filled out a survey on five consecutive workdays. In line with earlier day-level research (e.g., Sonnentag, Reinecke, Mata, & Vorderer, 2018), we opted for a data collection period of one workweek. On the first day (i.e., Monday), respondents needed to fill in an extended questionnaire to assess demographic variables and the trait variable empathy in addition to the daily measures of work-related smartphone use during off-job hours, workplace telepressure during off-job hours and psychological detachment.

Next, participants received an email on each of the remaining workdays (i.e., Tuesday until Friday) within that workweek as well. Emails were sent at approximately 17:30 p.m. and contained general instructions together with the URL to the questionnaire, which solely assessed the daily measures. In the general instructions, we emphasized that statements with regard to ICT-related behaviour and cognition referred to a highly specific context (i.e., work-related smartphone communication during off-job time) and that the daily survey should not be filled in immediately, but be postponed until bedtime. A kind reminder was sent at approximately 23:00 p.m. to participants who had not yet completed the survey. Data collection took place in the Netherlands. As such, communication and questionnaires of the current study were all in the native language, Dutch.

Of the 82 respondents who initially participated, two (2.4%) were removed from the analysis since they did not respond to at least four questionnaires on successive working days. In total, 45 participants (56.2%) filled out surveys for four workdays and 35 participants (43.8%) completed all surveys during the entire workweek. Presenting the majority, 50 participants (62.5%) were male. Mean age in the sample was 41 years (*SD* = 11.75) and mean organizational tenure was 11.5 years (*SD* = 9.95). Among all participants, 87.5% were full-time employed, 5% were part-time employed and 7.5% were self-employed. Also, 73.8% of the employees possessed a company-issued smartphone. A wide variety of occupations were represented, comprising the industrial sector (35%), real estate (18.4%), health care (13.8%) and education (8.8%).

### Trait measure

*Empathy* was measured using the other-oriented subscales of the Interpersonal Reactivity Index (Davis, 1983). The 7-item empathic concern subscale reflected the affective component of empathy. Example item read: ‘Sometimes I do not feel very sorry for other people when they are having problems (reversed item)’. The 7-item perspective taking subscale reflected the cognitive component of empathy. Example item read: ‘When I am upset at someone, I usually try to put myself in his/her shoes for a while’. All items were rated on a seven-point Likert scale ranging from 1 (*totally disagree*) to 7 (*totally agree*). As originally intended (Davis, 1983) and as supported by recent construct validity research on the IRI (Chrysikou & Thompson, 2016), the subscales of this instrument should not be combined together to form an overall measure of empathy. Accordingly, empathic concern and perspective taking were computed separately. Cronbach’s α of the subscales were, respectively, .77 and .72.

### State measures

*Daily workplace telepressure during off-job hours* was measured using the six-item Workplace Telepressure Measure developed by Barber and Santuzzi (2015). In line with prior research on telepressure (Barber & Santuzzi, 2017), we modified the original instrument for the purpose of the present study by altering its instructions. The adapted instrument is context-specific and assesses cognitions during off-job hours relating to business communication by means of the smartphone. Items were adjusted to day-level measurement by adding ‘Today …’ to each item. Example items read: ‘Today, I felt a strong need to respond to others immediately’ and ‘Today, it was hard for me to focus on other things when I received a message from someone”. All items were rated on a seven-point Likert scale ranging from 1 (*totally disagree*) to 7 (*totally agree*). Cronbach’s α of the scale varied between .83 and .92, with an average of .88 over all five research days.

*Daily work-related smartphone use during off-job hours* was measured using the four-item Smartphone Use Scale developed by Derks and Bakker (2014). Via the instructions, we emphasized that items exclusively addressed work-related smartphone use during off-job hours. Items were adjusted to day-level measurement by adding ‘Today …’ to each item. Example items read: ‘Today, I used my smartphone intensively for work-related purposes’ and ‘Today, I was online for work with my smartphone until I went to sleep’. All items were rated on a seven-point Likert scale ranging from 1 (*totally disagree*) to 7 (*totally agree*). Cronbach’s α of the scale varied between .49 and .75, with an average of .64 over all five research days.

*Daily psychological detachment from work* was measured using the four-item subscale of the Recovery Experiences Questionnaire (Sonnentag & Fritz, 2007). Items were adjusted to day-level measurement by adding ‘Today, in my free time after work …’ to each item. Example items read: ‘Today, in my free time after work, I forgot about work’ and ‘Today, in my free time after work, I distanced myself from my work’. All items were rated on a five-point Likert scale ranging from 1 (*totally disagree*) to 5 (*totally agree*). Cronbach’s α of the scale varied between .89 and .93, with an average of .91 over all five research days.

*Daily stress* was measured as a control variable because high levels of stress is potentially related to psychological detachment and may act as confounding variable. It was measured using the four-item version of the Perceived Stress Scale (Cohen, Kamarck, & Mermelstein, 1983). Items were adjusted to day-level measurement by adding ‘Today …’ to each item. An example item reads: ‘Today, how often have you felt difficulties were piling up so high that you could not overcome them?’. All items were rated on a six-point Likert scale ranging from 1 (*never*) to 6 (*always*). Cronbach’s α of the scale varied between .49 and .73, with an average of .63 over all five research days.

*Daily workload* was measured as a control variable because high workload is potentially related to psychological detachment and may act as confounding variable. It was measured using the three-item scale developed by Bakker, Demerouti, Taris, Schaufeli, and Schreurs (2003). Items were adjusted to day-level measurement by adding ‘Today …’ to each item. An example item reads: ‘Today, I had to work extra hard to finish things’. All items were rated on a six-point Likert scale ranging from 1 (*never*) to 6 (*always*). Cronbach’s α of the scale varied between .88 and .93, with an average of .91 over all five research days.

### Strategy of analysis

Since we included multiple measurements in our design, our data can be viewed as multilevel data, with repeated measurements nested within individuals. This results in a two-level model with the repeated measures (daily variables) at the first-level (*N* = between 336 and 400 study occasions) and the individual participants at the second level (*N* = 80 participants). We used multilevel analysis with the MLwiN program (Rasbash, Browne, Healy, Cameron, & Charlton, 2000) to analyze our data. Day-level variables—both predictor and control variables—were centered to the individual mean (Level 1: telepressure, work-related smartphone use, psychological detachment, workload and stress), and the person-level (Level 2) moderators (sub-dimensions of empathy) were centered to the grand mean (for a more detailed discussion on the centering of variables regarding cross-level effects, see Aguinis, Gottfredson, & Culpepper, 2013). In line with recent best-practice recommendations (Aguinis & Bernerth, 2016), control variables were only included when they were theoretically relevant and significantly related to the outcome variable.

## Results

### Descriptive statistics

Table 1 presents the means, standard deviations and correlations among the demographic, control and study variables. In order to examine the proportion of variance that is attributed to the different levels of analysis, we calculated the intra-class correlation (ICC1) for each day-level variable. Results showed that 50% of the variance of telepressure, 42% of the variance in work-related smartphone use during off-job hours, 52% of the variance in psychological detachment, 51% of the variance in workload and 45% of the variance in stress was attributable to within-person variations.

**Table 1:** Means, Standard Deviations, and Correlations for all Study Variables.

	M	SD	1.	2.	3.	4.	5.	6.	7.	8.

1. Gender	—	—								
2. Age	41.09	11.69	–.04							
3. Stress	2.11	.68	.10	–.08						
4. Workload	3.59	1.22	–.01	.06	.15**					
5. Workplace telepressure	3.20	1.27	.03	–.07	.12*	.15**				
6. Work-related smartphone use	3.53	1.29	.01	–.01	.04	.08	.29**			
7. Psychological detachment	4.24	1.60	–.09	–.07	–.16**	–.15**	–.16**	–.57**		
8. Empathic concern	4.85	.88	.09	.13**	.17**	.11	.22**	.20**	–.33**	
9. Perspective taking	4.93	.76	–.09	–.11*	.00	.19**	.08	.08	.03	.33**

*Note*: Gender (0 = male, 1 = female); M = mean; SD = standard deviation; correlations between and alpha estimates of daily variables are based on averaged scores across the five days that the study took place; *n* = 80 persons, and *n* = 336–400 occasions. ** *p* < .01, * *p* < .05.

### Hypotheses testing

Hypothesis 1 proposed that daily workplace telepressure during off-job hours will be negatively related to daily psychological detachment. We compared two models for daily psychological detachment: a predictor model with only the control variables (workload and stress) and a predictor model in which daily workplace telepressure was added. Results (see Table 2) showed that the predictor model explained no additional variance compared to the model only including the control variables (Δ–2x log = .91, *df* = 2, *p* = *ns*). The relation between telepressure and psychological detachment was non-significant (γ = –.077, SE = .08, *t* = .96, *p* = *ns*). Therefore, Hypothesis 1 had to be rejected.

**Table 2:** Multilevel Results of the Relation Between Daily Workplace Telepressure and Daily Psychological Detachment.

	Psychological detachment			

	Control model		Predictor model	

	*Estimate*	*Std. er.*	*Estimate*	*Std. er.*

Intercept	4.22***	.14	4.22***	.14
Workload	–.15	.08	–.14	.08
Stress	–.05	.16	–.04	16
Workplace telepressure			–.08	.08
Variance level 2 (employee)	1.22 (48%)	.25	1.23	.25
Variance level 1 (day)	1.32 (52%)	.12	1.13	.12
–2 Log likelihood	1172.744		1171.834	

*Note*: Data points = 336 of 400 cases in use (respondents *n* = 80, days *n* = 5). *** *p* < .001.

In Hypothesis 2, we predicted that employees would use their smartphone more intensively for work-related purposes during off-job hours on days that they experience more workplace telepressure during off-job hours. The multilevel model that contained daily telepressure as the predictor of daily work-related smartphone use was compared to the null model that included only the intercept (see Table 3). The model containing telepressure as a predictor showed a significant improvement over the null model (Δ–2x log = 9.4, *df* = 1, *p* < .005). The estimate of telepressure (γ = .176, *SE* = .06, *t* = 3.09, *p* < .001) was significant and positive, supporting Hypothesis 2: on days that employees experience more telepressure during off-job hours, they use their smartphones more intensively for work-related purposes during off-job hours.

**Table 3:** Multilevel Results of the Relation Between Daily Workplace Telepressure and Daily Work-related Smartphone-Use.

	Work-related smartphone use			

	Null model		Predictor model	

	*Estimate*	*Std. er.*	*Estimate*	*Std. er.*

Intercept	3.53	.12	3.53	.12
Workplace telepressure			0.18***	0.06
Variance level 2 (employee)	.98 (59%)	.18	0.98	.18
Variance level 1 (day)	.69 (41%)	.06	0.67	.06
–2 Log likelihood	986.358		976.956	

*Note*: Data points = 336 of 400 cases in use (respondents *n* = 80, days *n* = 5). *** *p* < .001.

According to Hypothesis 3, daily work-related smartphone use during off-job hours will be negatively related to daily psychological detachment. To test the hypothesis, we compared two models for daily psychological detachment: a predictor model with only the control variables (workload and stress) and a predictor model in which daily work-related smartphone use was added. Regarding the relation between work-related smartphone use and psychological detachment, results (see Table 4) showed that smartphone use was significantly and negatively related to psychological detachment (γ = –.48, *SE* = .08, *t* = 5.93, *p* < .001). Furthermore, the predictor model showed a significant improvement in explained variance over the model only including the control variables (Δ–2xlog = 31.83, *df* = 2, *p* < .001). This implies that Hypothesis 3 is supported. On days that employees use their smartphones more intensively for work during off-job hours, they detach less from work and job-related issues within the same day.

**Table 4:** Multilevel Results of the Relation Between Daily Work-related Smartphone Use and Daily Psychological Detachment.

	Psychological detachment			

	Control model		Predictor model	

	*Estimate*	*Std. er.*	*Estimate*	*Std. er.*

Intercept	4.22***	.14	4.22***	.14
Workload	–.15	.08	–.10	.08
Stress	–.05	.16	–.07	.15
Work-related smartphone use			–.48***	.08
Variance level 2 (employee)	1.22 (48%)	.25	1.26	.25
Variance level 1 (day)	1.32 (52%)	.12	1.16	.10
–2 Log likelihood	1172.744		1140.091	

*Note*: Data points = 336 of 400 cases in use (respondents *n* = 80, days *n* = 5). *** *p* < .001.

Subsequently, in the fourth hypothesis we proposed that the relationship between daily workplace telepressure during off-job hours and daily psychological detachment is mediated by daily work-related smartphone use during off-job hours. To test whether results indeed imply a mediation model, we first tested whether telepressure and psychological detachment were directly related (Mathieu & Taylor, 2006). Results showed that there is no significant direct relation between daily telepressure and daily psychological detachment (see above: rejection of Hypothesis 1). However, according to Mathieu and Taylor (2006) mediation inferences are justified when both the predictor-mediator and mediator-outcome paths are significant (see also Kenny, Kashy & Bolger, 1998; MacKinnon, Lockwood, Hoffman, West, & Sheets, 2002). Their operationalization states indirect effects as a special form of intervening effects where the predictor and outcome variable are not related directly, but they are indirectly related through significant relationships with a linking mechanism. Our results already established the significant relationships between telepressure and smartphone use (Hypothesis 2) and between smartphone use and psychological detachment (Hypothesis 3). Since both telepressure (independent variable) and psychological detachment (dependent variable) were significantly related to smartphone use (intervening variable), we performed a Sobel test (Sobel, 1982) to see whether the proposed indirect effect is significant (see Tables 3 and 4 for Sobel test input). In line with Hypothesis 4, results from the Sobel test support an indirect link between telepressure and psychological detachment via work-related smartphone use during off-job hours (*z* = –2.74, *p* < .01).

Regarding Hypothesis 5, we tested whether empathy (represented by the two sub-dimensions: empathic concern and perspective taking) moderates the positive relation between workplace telepressure and work-related smartphone use, such that the positive relation between workplace telepressure during off-job hours and work-related smartphone use during off-job hours will be stronger for employees who score high on empathy than for employees who score low on empathy. First we compared the predictor-only model, containing both empathic concern and telepressure with the interaction model adding the interaction term of telepressure and empathic concern (see Table 5). The interaction model showed no significant improvement in explained variance over the model only including the predictor variables (Δ–2xlog = 2.22, *df* = 1, *p* = *ns*). The estimate of the interaction between telepressure and empathic concern was non-significant (γ = –.098, *SE* = .07, *t* = –1.48, *p* = *ns*). Next, we tested whether the perspective taking dimension of empathy moderates the relation between daily workplace telepressure during off-job hours and daily work-related smartphone use during off-job hours. Again, we compared the predictor-only model—including telepressure and perspective taking—with the interaction model in which the interaction between telepressure and perspective taking was added. Results (see Table 6) showed that the interaction model explained no additional variance compared to the predictor-only model (Δ–2xlog = .04, *df* = 1, *p* = *ns*). The estimate of the interaction between telepressure and perspective taking was non-significant (γ = .015, *SE* = .08, *t* = 3.05, *p* = *ns*). Therefore Hypothesis 5 had to be rejected.

**Table 5:** Multilevel Results of the Interaction of Empathic Concern and Daily Telepressure on Daily Work-related Smartphone Use.

	Work-related smartphone use			

	Predictor-only model		Interaction model	

	*Estimate*	*Std. er.*	*Estimate*	*Std. er.*

Intercept	3.53***	.12	3.53***	.12
Workplace telepressure	.18**	.06	.18**	.06
Empathic Concern	.30*	.13	.29*	.13
Concern x Telepressure			–.10	.07
Variance level 2 (employee)	.91 (58 %)	.17	.92	.17
Variance level 1 (day)	.67 (42 %)	.06	.66	.06
–2 Log likelihood	972.019		969.802	

*Note*: Data points = 336 of 400 cases in use (respondents *n* = 80, days *n* = 5). *** *p* < .001, ** p < .01, * p < .05.

**Table 6:** Multilevel Results of the Interaction of Perspective taking and Daily Telepressure on Daily Work-related Smartphone Use.

	Work-related smartphone use			

	Predictor-only model		Interaction model	

	*Estimate*	*Std. er.*	*Estimate*	*Std. er.*

Intercept	3.53***	.12	3.53***	.12
Workplace telepressure	.18**	.06	.17**	.06
Perspective taking	.17	.16	.17	.16
Perspective x Telepressure			.02	.08
Variance level 2 (employee)	.97 (59 %)	.18	.97	.18
Variance level 1 (day)	.67 (41 %)	.06	.67	.06
–2 Log likelihood	975.850		975.810	

*Note*. Data points = 336 of 400 cases in use (respondents *n* = 80, days *n* = 5). *** *p* < .001, ** *p* < .01.

## Discussion

Literature on recovery and mental health has identified psychological detachment from work as a crucial and powerful experience to recover from work demands and provided ample evidence that this experience can affect employees’ well-being and performance capabilities (e.g., Etzion et al., 1998; Sonnentag & Bayer, 2005). Unfortunately, the introduction of ICT devices into the workplace can make it more difficult to mentally disconnect from work. Indeed, employees’ psychological detachment seems to be hindered by work-related ICT use during off-job hours such as replying to work-related messages (Derks et al., 2014; Schlachter et al., 2018; Van Laethem et al., 2018) and by the corresponding preoccupation with and urge for sending those replies quickly, which is also referred in literature as workplace telepressure (Barber & Santuzzi, 2015; Grawitch et al., 2018; Santuzzi & Barber, 2018).

Building on this line of research, the purpose of the present study was to investigate the direct effect of employee’s workplace telepressure during off-job hours on their psychological detachment and whether work-related smartphone use during off-job hours has a mediating role in the relationship. In addition, we aimed to explore whether individual differences in empathy play a moderating role between workplace telepressure and work-related smartphone use. A diary approach was conducted to provide insight into the daily fluctuations of our study variables. Diary designs generate more reliable and valid data, especially when compared to traditional survey designs, due to a decrease in respondents’ retrospective bias as data is collected close to the actual experience (Bolger, Davis, & Rafaeli, 2003). Additionally, the explained variance on the day-level was high in our sample, which supports the finding that work stressors and recovery levels may fluctuate from day to day (Ohly et al., 2010).

While previous studies have examined telepressure over longer periods of time (Barber & Santuzzi, 2017; Santuzzi & Barber, 2018), shorter-term trends have not been addressed up to now. Compared to longitudinal designs with time lags of several months, the diary approach is excellent for capturing short-term temporal dynamics of a variable of interest and provides additional information about the stability of the construct (Bolger et al., 2003). In line with the longer-term findings, the intra-class correlation coefficient (ICC1) for workplace telepressure revealed approximately equal amounts of within-person and between-person variability. Thus, an important share of the variance in workplace telepressure is located at the within-person level, which indicates that besides a general consistency of the construct, workplace telepressure also fluctuates substantially within the individual across situations and time. Our findings align with recent research which states that many applied psychology constructs do not only vary between individuals, but also vary within the individual (Podsakoff, Spoelma, Chawla, & Gabriel, 2019).

By examining workplace telepressure more precisely in a leisure context, we were able to determine its impact on work recovery in an ecologically valid manner (Judge & Kammeyer-Mueller, 2012). However, contrary to our expectation, workplace telepressure during off-job hours was at day-level not significantly related to psychological detachment. This finding further supports the notion of Grawitch and colleagues (2018) that workplace telepressure does not interfere with the off-job experience of disconnecting mentally from work. Interestingly, there is also longitudinal evidence that workplace telepressure is negatively related to psychological detachment (Santuzzi & Barber, 2018), which is in contrast with our finding. One possible explanation for these conflicting findings might be the choice of time lags between measurement periods. Furthermore, it should be noted that the experience of workplace telepressure might differ between work and nonwork hours. If so, the construct of telepressure in the study of Santuzzi and Barber (2018) is being too generic and potentially misleading for precise theory testing.

In support of our second hypothesis, workplace telepressure during off-job hours appeared to have a small positive effect on work-related smartphone use during off-job hours on a day-to-day basis. This is in line with the study of Van Laethem and colleagues (2018) in which workplace telepressure, assessed as a generic and between-person measure, was found to be a significant predictor of work-related smartphone use during and after work hours. Considering email as one of the mostly used communication channels on the smartphone (Steemers et al., 2017), our finding also nicely confirms the assumption that workplace telepressure may elicit a more frequent email response (Barber & Santuzzi, 2015). However, our finding and those of Van Laethem and colleagues (2018) is inconsistent with earlier research on email responsiveness after work hours (Grawitch et al., 2018). Cross-sectional evidence by Grawitch and colleagues (2018) indicated that workplace telepressure did not explain additional variance in business email response frequency after work hours beyond personal and organizational factors. It might be plausible that during off-job hours workplace telepressure triggers a more general and a less time consuming use of the smartphone such as checking for work-related ICT messages or being available for work-related issues, instead of actually replying to these messages.

As expected, we replicated the finding that daily fluctuations in work-related smartphone use during off-job hours was moderately negatively related to daily fluctuations in psychological detachment (Derks et al., 2014; Van Laethem et al., 2018), which further supports prior research findings that link work-related ICT use at home to a lack of employees’ capability to mentally distance themselves from work (Park et al., 2011; Schlachter et al., 2018). Inspired by the effort-recovery theory of Meijman and Mulder (1998), this finding reflects that fluctuations in smartphone use for work-related purposes during off-job hours (e.g., at home) over extended periods of time (i.e., consecutive workdays) may become a fluctuating stressor or a fluctuating demanding and energy consuming activity, impairing one’s psychological detachment from work.

At first sight, the mediation hypothesis indicating that daily workplace telepressure during off-job hours would be related to daily psychological detachment via daily work-related smartphone use during off-job hours had to be rejected because workplace telepressure and psychological detachment were, at day-level, not directly related (Mathieu & Taylor, 2006). However, both workplace telepressure and psychological detachment were meaningfully related to work-related smartphone use, which is consistent with an indirect effect inference as argued by Mathieu and Taylor (2006). More specifically, our result suggests that workplace telepressure during off-job hours slightly elicits work-related smartphone behaviour during off-job hours, which in turn, obstructs employees’ psychological detachment from work in a moderate manner. In other words, the relation between two cognitive states/processes (i.e., daily workplace telepressure and daily psychological detachment) is indirect and can be explained through actual behaviour (i.e., daily work-related smartphone use). The mere preoccupation and urge to timely respond to work-related ICT messages during off-job hours does not interfere with employees’ detachment from work. However, psychological detachment can be obstructed when the employee gives in to this desire or urge by actually utilising their smartphone for work-related purposes during off-job hours. The indirect relation is in line with cognitive psychological theory (Bandura, 1986), which states that cognitive processes often precede human behaviour.

In relation to our final hypothesis, we did not find a moderating effect of employees’ other-oriented empathy on the relationship between daily workplace telepressure during off-job hours and daily work-related smartphone use during off-job hours. It seems that neither the affective component (i.e., empathic concern) nor the cognitive component (i.e., perspective taking) strengthened the positive relationship. These results imply that employees’ other-oriented empathy does not reinforce their urge to timely respond to work-related ICT messages. However, empathic concern had a small positive main effect on work-related smartphone use during off-job hours, a finding which provides further evidence that feelings of empathy also play a role in computer-mediated communication (Carrier et al., 2015). Furthermore, this finding is consistent with previous work regarding the impact of other-oriented empathy on helping behaviour (Eisenberg et al., 2006).

### Limitations

The presented results need to be interpreted with caution, owing to some study limitations. First and foremost, it should be noted that all state measures, which were assessed on a daily basis, were collected at the same time (i.e., the moment just before going to bed). Therefore, the temporal order of the study variables could not be established within our design, which prevents us from inferring causal statements.

Although we used a diary approach, which is currently the most popular method when it comes to the tracing of employees activity in everyday life (Ohly et al., 2010), our study variables are exclusively self-reported, which can give rise to concerns of common-method bias (Podsakoff, MacKenzie, Lee, & Podsakoff, 2003). However, we tried to minimize this concern by using existing valid scales with good internal consistencies and consisting of multiple items (Spector, 2006) and emphasized that participants’ responses were anonymously processed. Furthermore, in our multilevel approach, we were primarily interested in intraindividual fluctuations over days, which eliminates the potential influence of response tendencies stemming from individual differences and thus further reduces issues associated with common-method variance.

As the present study addressed a day-level perspective to shed light on the within-person fluctuations within telepressure over time that has been heretofore exclusively limited to a month-level approach (Barber & Santuzzi, 2017; Santuzzi & Barber, 2018), our study is not justified to infer statements beyond this time lag. For instance, poor psychological detachment during a few days will likely lead to temporary experiences at most. Failing to detach within a given day is therefore not as problematic as a chronic lack of detachment due to repeated encounters of job stressors, which in turn may have longer-term decrements in well-being (Sonnentag, Binnewies, & Mojza, 2010).

Next, our measurement scales for work-related smartphone use during off-job hours and stress showed, on certain days, poor Cronbach’s alpha values. Although abbreviated and adapted scales in daily assessments are recommended to decrease total survey length (Reis & Gable, 2000), these scales often have lower reliability estimates as Cronbach’s alpha is highly sensitive to the number of items in the scale (Pedhazur & Schmelkin, 1991). Indeed, psychometric properties of the original Perceived Stress Scale (Cohen et al., 1983) have repeatedly shown to be superior to those of the abbreviated scale (Lee, 2012). Analogous to the Perceived Stress Scale, the Cronbach’s alpha estimates of the Smartphone Use Scale (Derks & Bakker, 2014) were presumably also affected by the small number of items that tap into slightly different aspects of smartphone use. Additionally, the changes in wording of the items to work-related use and daily experience may have enhanced the risk of relatively low scale reliability. Although, using a daily diary design, Van Laethem and colleagues (2018) did report satisfactory alpha estimates of the adapted scale.

A final limitation pertains to the sample size being limited to 80 participants. Whereas this relatively small sample size is in line with recommendations regarding the bare minimum sample size for multilevel modelling (i.e., 50 level-two observations; Kreft & de Leeuw, 1998), one still should be cautious to generalize the current findings to the whole population of employees as sampling variability may be substantial. Therefore, we encourage future studies to attempt to replicate these results with a larger sample, as well as by taking into account the other above-mentioned limitations.

### Implications for practice

Besides theoretical contributions to literature on the impact of ICTs on recovery and mental health, our findings are also valuable in practice as smartphones enable employees to communicate with clients and colleagues regardless of time or place. Although employees may think that quickly responding to a work-related message after work is harmless and innocent, our results provide empirical evidence for the opposite. We found that workplace telepressure elicited work-related smartphone use on a day-to-day basis. Within the same day, this ultimately translated into a diminished psychological detachment from work, which over time may turn into a chronic state.

Thus, we would recommend that organizations develop a good practice to aid their employees in detaching from work by, for example, creating a shared awareness of ICT-related health risks and clearly informing them about the organization’s response expectations towards business communication. Organizational policies can be applied to encourage employees not to contact colleagues during off-job hours, and if doing so, one could be enforced to explicitly mention in their communications that a response does not need to be quickly sent, but can wait until the next workday. After all, maintenance of good employee mental health is also of great interest to the employing organization, since it relates to increased business productivity and decreased organizational costs (Harter et al., 2003).

At the individual level, there are several strategies that employees can use. Our results suggest that strategies focussing exclusively on behavioural change may already be sufficient to foster psychological detachment, considering it is only the conversion of one’s intention to respond to a work-related ICT message into real action that undermines detachment from work. Drastic measures such as putting your smartphone away or leaving it behind at the workplace may not be required. By changing certain inbox settings or downloading specific smartphone applications, one can simply block and withheld popup notifications of work contacts during leisure time, which can lessen or even completely diminish work-related smartphone use after work.

## Conclusion

Our diary study aimed to further elucidate the impact of workplace telepressure on psychological detachment from work. Our findings have shown that the experienced workplace telepressure during off-job hours obstructs psychological detachment only in an indirect way through work-related smartphone behaviour during off-job hours, implying that employees’ recovery processes might become undermined. Furthermore, the positive relationship between workplace telepressure and smartphone use for work purposes existed irrespective of employees’ other-oriented empathy. These results provide practitioners and employers with new insights on how to foster psychological detachment and further illustrate the importance of proper smartphone use among employees.
